# Extradural Dermoid Cyst of Mastoid Bone: A Case Report

**DOI:** 10.1155/2012/548340

**Published:** 2012-08-15

**Authors:** Hamad S. Al-Muhaimeed, Hazem Y. Abdelwahed, Essam A. Elgamal, Ghassan M. Alokby, Ameen M. Binnasser, Masoada M. Ashraf

**Affiliations:** ^1^Department of Otorhinolaryngology, King Abdul Aziz University Hospital, College of Medicine, King Saud University, Riyadh, Saudi Arabia; ^2^Neurosurgery Division, King Khalid University Hospital, College of Medicine, King Saud University, Riyadh, Saudi Arabia

## Abstract

Dermoid cysts of the head and neck are rare congenital benign tumors. According to the literature they represent about seven percent of all dermoids and less than one percent of all intracranial neoplasms. Extradural dermoid cysts are very rare. We report a case of intracranial extradural dermoid cyst of mastoid bone. We believe that this is the second documented extradural dermoid cyst, the first case reported in the literature (Ammirati et al., 2007) was in close relation to the petrous apex but ours is in close relation to mastoid antrum. Hearing loss was the only clinical presentation in this case, while neurological symptoms were the main presenting symptoms in the first reported case. We present our management of this rare case with respect to the clinical, radiological, histopathological, and surgical aspects and conclude that dermoid tumors, though rare, need to be included in differential diagnosis of middle ear lesions.

## 1. Introduction

Dermoid tumors are not true neoplasms but are inclusion cysts composed of ectodermal elements. They are uncommon lesions, accounting for approximately 0.3% of all brain tumors and about seven percent of all dermoids of the head and neck [1].

Dermoid cysts are derived from both the ectodermal and mesodermal elements. A keratinizing squamous epithelium is typically present along with dermal derivatives including hair follicles, smooth muscle, and apocrine and sebaceous glands. Fibroadipose tissue is also present. The exact etiology of these neoplasms is unknown though the most likely theories are incomplete closure at lines of fusion or traumatic implantation of skin elements [2]. 

We report a case of intracranial extradural dermoid cyst of mastoid bone, with respect to the clinical, radiological, histopathological, and surgical aspects.

## 2. Case Report

An 18-years-old girl diagnosed at the age of three years to have right otitis media with effusion (OME) was treated medically. She presented again at the age of 8 years with the same complaint but did not respond to the medical treatment and underwent right myringotomy with ventilation tube and adenoidectomy. Two years later, her symptoms recurred and underwent right T-tube insertion. Symptoms had recurred again after removal of the T-tube 2 years later.

She presented at the age of 18 years with right hearing loss and intermittent nonpulsatile tinnitus. There was no history of otorrhoea, otalgia, or vertigo, and there were no abnormal neurological signs or symptoms.

**Figure 1:**
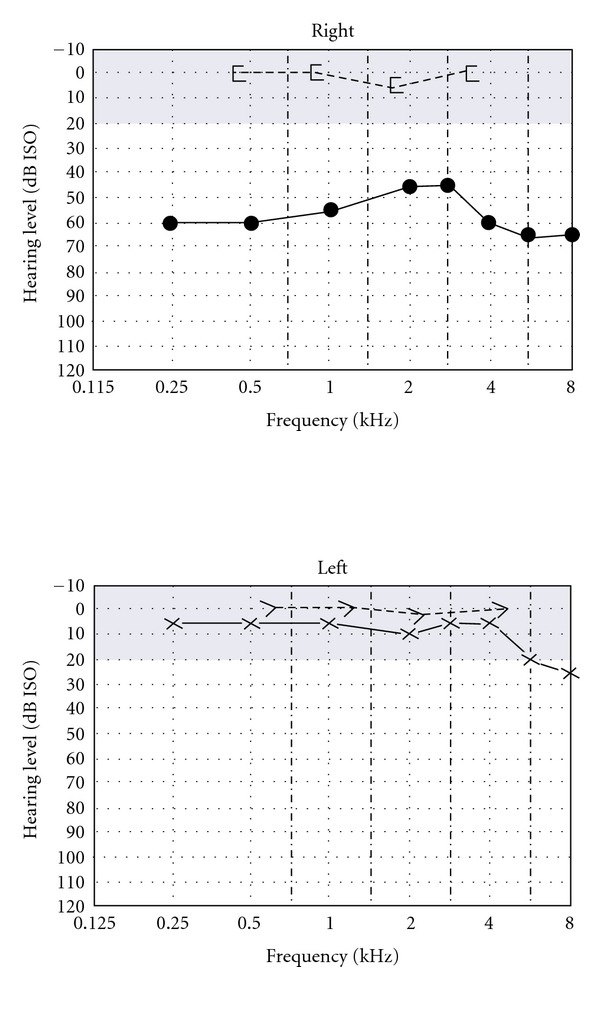
Audiogram showing right mild to moderate conductive hearing loss with air bone gab, accounting from 40 to 60 dB loss and normal left hearing level.

Physical examination revealed retracted right tympanic membrane, while the left was normal. Audiogram showed right moderate conductive hearing loss (CHL) (Figure 1) with shallow type-A tympanogram. 

**Figure 2:**
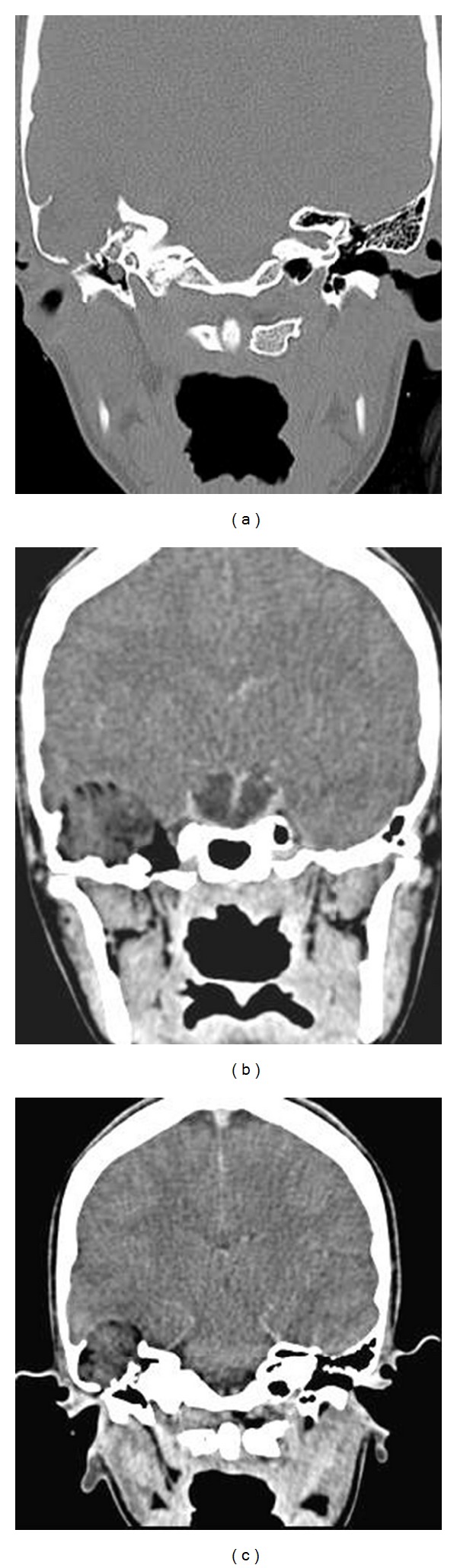
Computed tomography: coronal image, bone window (a) and soft tissue window with contrast (b and c) showing sclerotic contracted right mastoid temporal bone with most mastoid air cells being occupied by polypoidal expansile soft tissue densification lesion (heterogeneous, nonenhanced in contrasted soft tissue window image).

Computed tomography (CT) of the temporal bone (Figure 2) showed right mastoid and middle ear mass with intracranial extradural extension elevating the right temporal lobe of the brain. The lesion was associated with marked erosion of the floor of middle cranial fossa.

**Figure 3:**
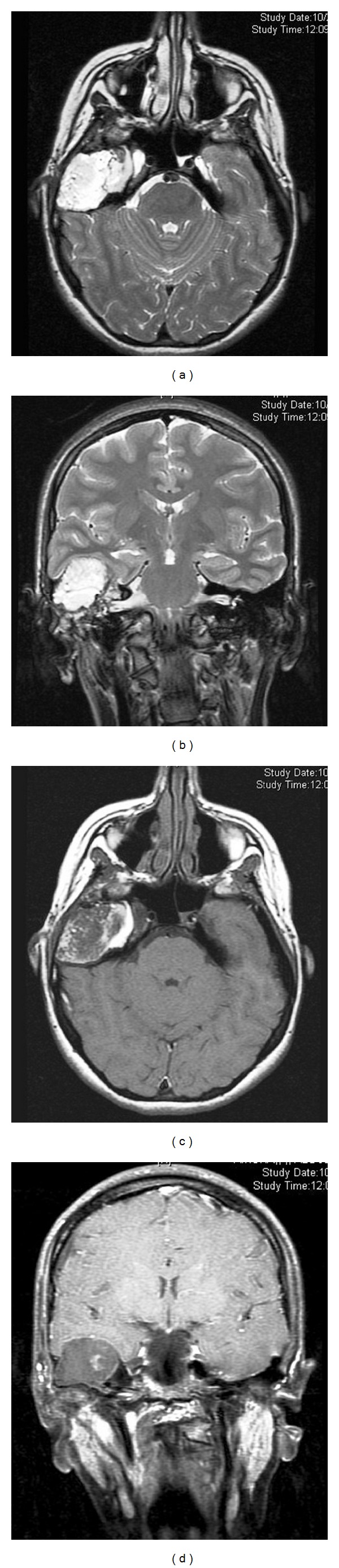
Axial T2-WI (a), coronal T2WI (b), axial T1-Gd (c), and coronal T1-Gd (d) showing a large expansile extraaxial right mastoid mass causing elevation of the right temporal lobe demonstrate heterogeneous signal intensity on T2 and minimal enhancement in post contrast.

Magnetic resonance imaging (MRI) of the temporal bone and brain with and without gadolinium (Figure 3) revealed T1 and T2 sequences of heterogeneous but predominantly hyperintense mass located mainly in the right mastoid and middle ear cavity causing elevation of the right temporal lobe of the brain. Initial diagnosis of congenital cholesteatoma with erosion of floor of middle cranial fossa and extracranial extension was made.

The patient underwent a combined approach: right temporal craniotomy with middle cranial fossa approach to excise this mass by the neurosurgeon, associated with canal wall down mastoidectomy by the otologist, which was necessary to eradicate the whole mass. The mass was encapsulated and adherent to the dura of the middle cranial fossa. Incision of the mass produced yellowish cheesy content with hair. A whitish mass was occupying the antrum and mastoid air cells. No incus or stapes suprastructure was seen, and the proper middle ear cavity was intact. The patient tolerated the procedure well and recovered with no complications.

**Figure 4:**
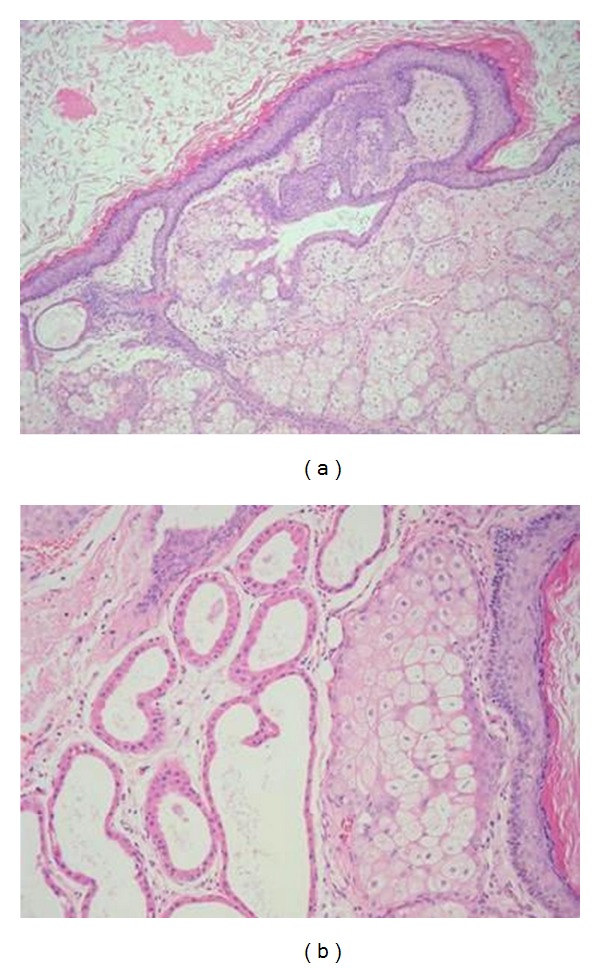
(a) Hematoxylin and Eosin stain (x100 magnification) section showing histology of a dermoid cyst. The cyst is filled with keratinous material in the lumen and is lined by stratified squamous epithelium that is responsible for the synthesis of luminal keratin. The tissue underlying the squamous lining comprises pilosebaceous units (b).

**Figure 5:**
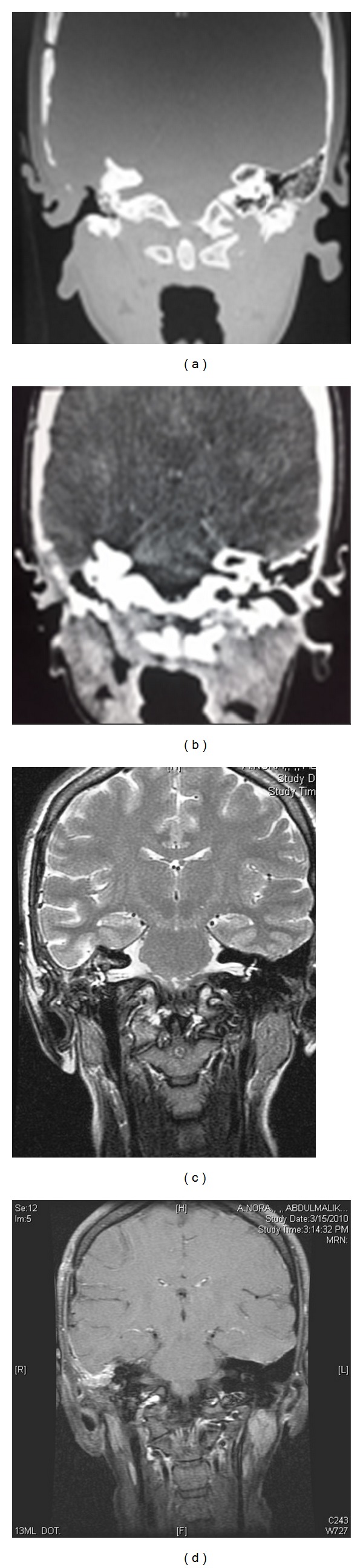
Three months after surgery, the CT (a, b) and MRI (c, d) of the temporal bone and brain revealed complete resection of the dermoid cyst and return of the right temporal lobe of brain to its normal position.

Histopathological report (Figure 4) showed dermoid cyst. Three months later, the CT with and without contrast and MRI with and without gadolinium of the temporal bone and brain (Figure 5) revealed complete resection of the dermoid cyst and return of the right temporal lobe of the brain to its normal position.

## 3. Discussion

Congenital dermoids are benign developmental anomalies rather than true neoplasms. They originate during early embryogenesis and are derived from both ectodermal and mesodermal elements (4). The exact etiology of these lesions is unknown, though the most likely theories are incomplete closure at lines of fusion or traumatic implantation of skin elements [3].

Dermoid cysts of head and neck are rare. They represent about 7% of all dermoids [3, 4] and less than 1% of all intracranial neoplasms [2, 4]. As reported by New and Erich [5], about 49.5% of head and neck dermoids are located in the periorbital region, 25% are located in the oral cavity, and 13% occur in the nasal cavity.

Twenty-four cases of dermoids of the temporal bone were reported in the English literature [6]. Multiple sites of involvement within the temporal bone have been described [1–4, 6]. The relationship of intracranial dermoid cyst to the dura matter was not emphasized, and intradural and interdural dermoids have been reported [4].

Grossly, dermoids are usually polypoid, pedunculated and rarely sessile masses. They are grayish white or pink in color, covered by skin often containing hair. Microscopically the surface epitheliod layer is of stratified squamous epithelium that contains epidermal appendages. The stroma is fibrofatty material and may contain smooth and stratified muscle, cartilage, bone, minor salivary glands, nerves, and lymph nodes.

Clinical presentation varies and usually depends on the location of the tumor. Dermoid tumors of the middle ear may present as unresolving serous otitis media or recurrent otitis media as in our case. This could be caused by the obstruction of the Eustachian tube by the dermoid. Negative pressure is created within the tympanic cavity and in mastoid air cells associated with low oxygen tension resulting in alteration in middle ear mucosa with an increase in mucus secreting cells.

The present case is unique because it represents extradural, intracranial dermoid tumor of mastoid antrum and was associated with well-defined osseous erosion. We believe that our case is the second documented case of extradural dermoid cyst. The first one was adjacent to the petrous apex [4].

Dermoid cysts appear hypodense on CT scan as a result of high lipid content. On MRI imaging dermoid tumors are hyperintense on T1-weighted sequences and are variable from hypo- to hyperintense on T2-weighted sequences due to their high fat content. Differential diagnosis of these tumors includes teratoma, temporal lobe meningocele herniating into mastoid antrum, meningiomas, lipoma, and congenital cholesteatoma.

The recommended treatment is complete surgical excision, and this can be achieved safely by the cooperation of multidisciplinary teams as in our case, the dermoid was totally excised without complications. These tumors have a limited growth potential and when completely removed they do not recur.

The prognosis of dermoids in the head and neck region is favorable. Malignant transformation in a long-standing dermoid cyst is a rare complication. Tsugu et al. [7] reported a case of squamous cell carcinoma (SCC) arising in an intracranial dermoid cyst. Devine and Jones [8] reported a case of malignant transformation to squamous cell carcinoma of a long-standing sublingual dermoid cyst.

## 4. Conclusion

The authors describe an unusual case of an extradural dermoid tumor within the mastoid antrum, and middle cranial fossa who presented with hearing loss proceeded by unresolving serous otitis media. The tumor was successfully and totally removed. Distinction from congenital cholesteatoma is impossible pre-operatively.

## References

[1] Smirniotopoulos JG, Chiechi MV (1995). Teratomas, dermoids, and epidermoids of the head and neck. *Radiographics*.

[2] Sturiale CL, Mangiola A, Pompucci A, D’Ercole M, Muro LD, Anile C (2009). Interdural giant dermoid cyst of the petrous apex. *Journal of Clinical Neuroscience*.

[3] Farris PE, Meyerhoff WL, Vuitch F (1998). Congenital dermoid cyst of the middle ear. *Skull Base Surgery*.

[4] Ammirati M, Delgado M, Slone HW, Ray-Chaudhury A (2007). Extradural dermoid tumor of the petrous apex. Case report. *Journal of Neurosurgery*.

[5] McAvoy JM, Zuckerbraun L (1976). Dermoid cysts of the head and neck in children. *Archives of Otolaryngology*.

[6] Kollias SS, Ball WS, Prenger EC, Myers CM (1995). Dermoids of the eustachian tube: CT and MR findings with histologic correlation. *American Journal of Neuroradiology*.

[7] Tsugu H, Fukushima T, Hayashi S, Iwaasa M, Matsuda T (2001). Squamous cell carcinoma arising in an intracranial dermoid cyst. *Neurologia Medico-Chirurgica*.

[8] Devine JC, Jones DC (2000). Carcinomatous transformation of a sublingual dermoid cyst. *International Journal of Oral and Maxillofacial Surgery*.

